# Very Early-Onset Inflammatory Manifestations of X-Linked Chronic Granulomatous Disease

**DOI:** 10.3389/fimmu.2017.01167

**Published:** 2017-09-26

**Authors:** Roxane Labrosse, Jane Abou-Diab, Annaliesse Blincoe, Guilhem Cros, Thuy Mai Luu, Colette Deslandres, Martha Dirks, Laura Fazilleau, Philippe Ovetchkine, Pierre Teira, Françoise LeDeist, Isabel Fernandez, Fabien Touzot, Helene Decaluwe, Ugur Halac, Elie Haddad

**Affiliations:** ^1^Department of Pediatrics, CHU Sainte-Justine, University of Montreal, Montreal, QC, Canada; ^2^Department of Microbiology, Infectiology and Immunology, CHU Sainte-Justine, University of Montreal, Montreal, QC, Canada

**Keywords:** chronic granulomatous disease, X-linked, gastric outlet obstruction, colitis, eosinophilia, *Mycobacterium avium*, early-onset

## Abstract

Chronic granulomatous disease (CGD) is a rare primary immune deficiency caused by mutations in genes coding for components of the nicotinamide adenine dinucleotide phosphate oxidase, characterized by severe and recurrent bacterial and fungal infections, together with inflammatory complications. Dysregulation of inflammatory responses are often present in this disease and may lead to granulomatous lesions, most often affecting the gastrointestinal (GI) and urinary tracts. Treatment of inflammatory complications usually includes corticosteroids, whereas antimicrobial prophylaxis is used for infection prevention. Curative treatment of both infectious susceptibility and inflammatory disease can be achieved by hematopoietic stem cell transplantation. We report herein three patients with the same mutation of the *CYBB* gene who presented with very early-onset and severe GI manifestations of X-linked CGD. The most severely affected patient had evidence of antenatal inflammatory involvement of the GI and urinary tracts. Extreme hyperleukocytosis with eosinophilia and high inflammatory markers were observed in all three patients. A *Mycobacterium avium* lung infection and an unidentified fungal lung infection occurred in two patients both during their first year of life, which is indicative of the severity of the disease. All three patients underwent bone marrow transplantation and recovered fully from their initial symptoms. To our knowledge, these are the first reports of patients with such an early-onset and severe inflammatory manifestations of CGD.

## Introduction

### Case 1

**Figure 1:**
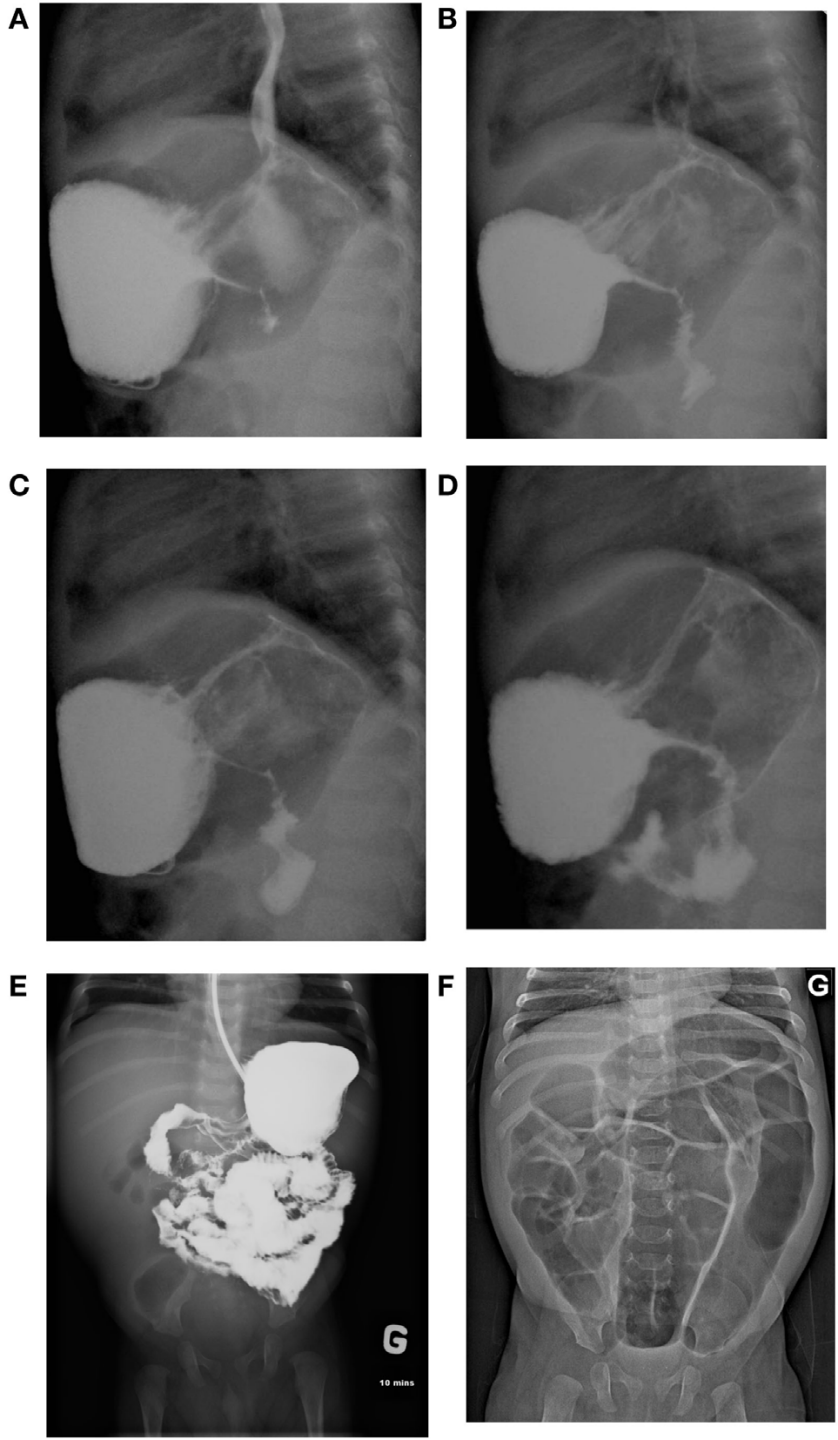
**(A–D)** Slowed gastric emptying and luminal thinness of antrum and pylorus on the barium meal (case 1). **(E)** Upper gastrointestinal (GI) series showing antropyloric stenosis with parietal and mesenteric infiltration of the duodenojejunal area (case 2). **(F)** Diffuse intestinal dilatation secondary to lower GI obstruction (case 2).

A 6-month-old French Canadian boy with a medical history of cow’s milk protein allergy was admitted to a community hospital for vomiting, feeding issues, and lethargy without fever. He had been on a hydrolyzed formula for cow’s milk protein allergy since the age of 4 months, following a history of feeding difficulties and associated rectal bleeding. Family history was unremarkable, except for infertility in the father. Both siblings (cases 1 and 2) were conceived from different sperm donors. The complete blood count revealed leukocytosis (WBC > 38.49 × 10^9^/L; 26.2 × 10^9^ neutrophils/L; 3.1 × 10^9^ eosinophils/L), an increased C-reactive protein (47 mg/L), and a normal sedimentation rate. The patient was referred to our center because of persistence of vomiting and leukocytosis (Table 1). The patient also presented with failure to thrive in the month preceding his admission, with a fall from the 50th percentile for weight down to the 15th percentile. An abdominal ultrasound (US) revealed major thickening of the pylorus up to the antrum and the gastric body. A barium meal showed delayed gastric emptying and luminal thinness of the antrum and pylorus (Figures 1A–D). Endoscopic biopsies of the esophagus and stomach revealed eosinophilic infiltration of the mucosa and submucosa. Bone marrow aspiration was normal. Whole-body positron emission tomography (PET) scan confirmed abnormal activity in the upper gastrointestinal (GI) tract. During his hospitalization, he developed a *Staphylococcus aureus* bacteremia 72 h following central line installation for parenteral feeding and was successfully treated with IV cloxacillin.

**Table 1:** Range of sequential laboratory data pre and post-corticosteroids.

	Case #1		Case #2		Case #3	
	Pre	On	Pre	On	Pre	On
WBC (×10^9^/L)	26.97–44.14	14.23–16.91	30.61–107.8	3.77–29.23	11.87–19.55	7.28
ANC (×10^9^/L)	14.8–26.7	1.2–5.3	10.47–63.6	2.9–16.4	5.9–6.7	2.0
ALC (×10^9^/L)	5.9–10.5	7.4–11.4	6.12–19.4	4.9–14.6	4.8–8.9	3.7
AEC (×10^9^/L)	1.3–6.8	0.4–1.0	2.45–12.9	0.1–1.4	0.5–0.9	0.1
CRP (mg/L)	47.7–147	–	51.3–89.4	0.2–29.3	10.9–28.7	4.4

*WBC, white blood cells; ANC, absolute neutrophil count; ALC, absolute lymphocyte count; AEC, absolute eosinophil count; CRP, C-reactive protein; Pre, pre-corticosteroids; On, per-corticosteroids*.

**Figure 2:**
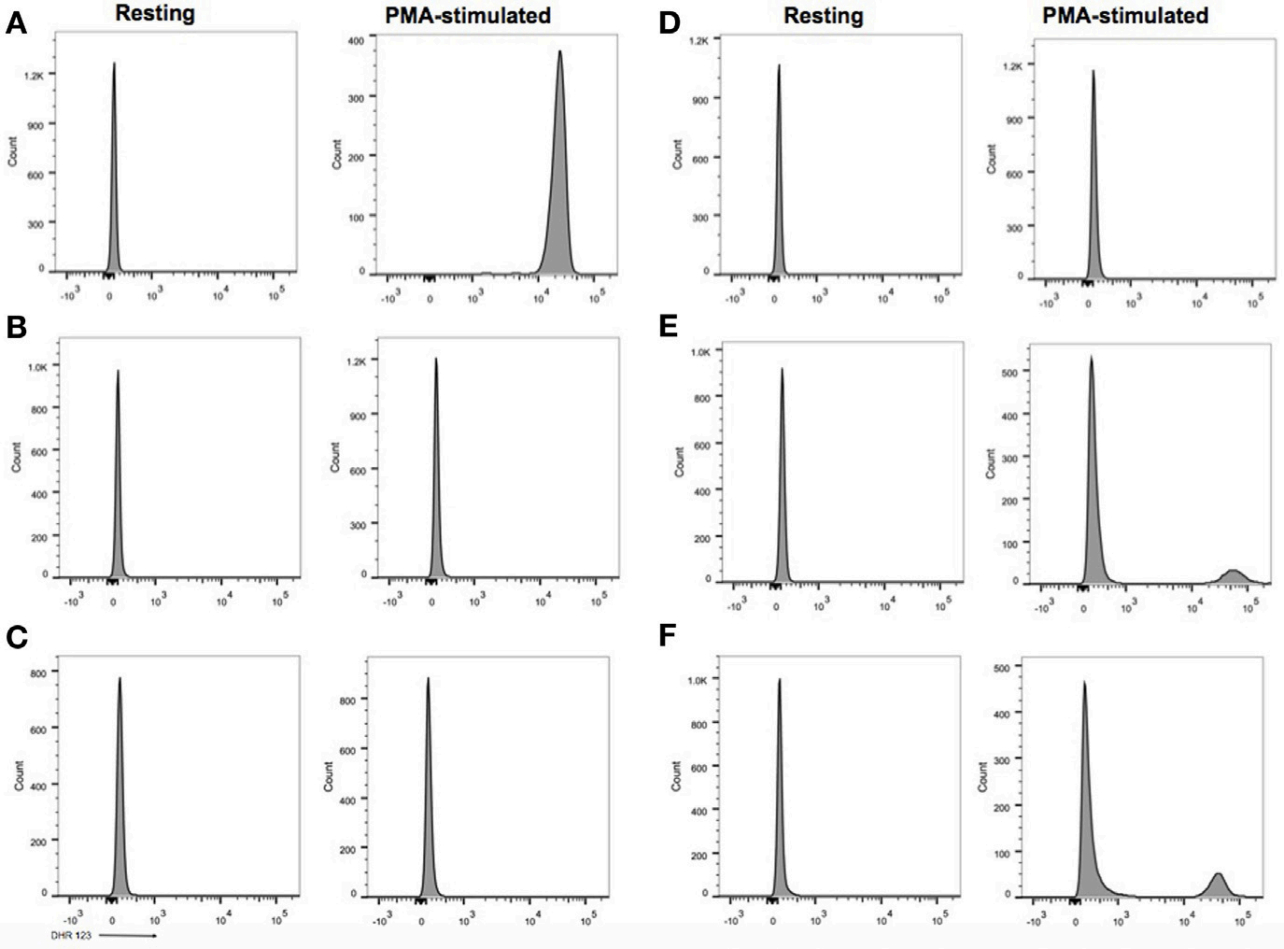
Flow cytometric dihydrorhodamine (DHR) 123 assay. **(A)** Healthy control, neutrophil stimulation index (SI): 305 (normal laboratory value > 91). **(B)** Case 1, SI: 1. **(C)** Case 2, SI: 1. **(D)** Case 3, SI: 1. **(E)** Mother of cases 1 and 2 with carrier status, SI: 1.6 for 86% of neutrophils, 391 for 14% of neutrophils. **(F)** Mother of case 3 with carrier status, SI: 2 for 80% of neutrophils, 252 for 20% of neutrophils.

**Figure 3:**
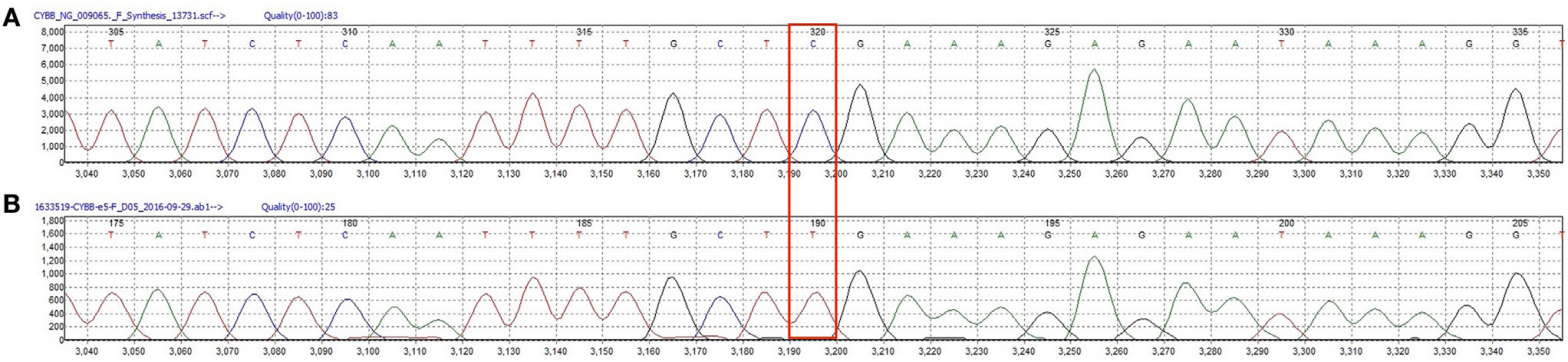
**(A)** Wild type CYBB sequence from a healthy donor. **(B)** Patients’ CYBB sequence featuring a c.496C>T point mutation leading to the p.Arg157* protein variant.

The diagnosis of chronic granulomatous disease (CGD) was confirmed by dihydrorhodamine (DHR) flow cytometry assay (Figure 2) and he was found to have a hemizygous nonsense mutation of the *CYBB* gene (c.469C>T mutation leading to a p.Arg157* protein variant) (Figure 3). DHR flow cytometry assay and genetic analysis on the mother confirmed her carrier status. Additional screening with thoraco-abdominal computerized tomography (CT) scan did not show granulomas or deep abscesses, but did reveal pulmonary ground-glass opacities. A bronchoalveolar lavage was subsequently performed and *Actinomyces* was isolated on culture. Prednisone therapy was initiated for the gastric inflammation, along with prophylactic cotrimoxazole and itraconazole, and therapeutic amoxicillin for the *Actimomyces*. The leukocytosis progressively normalized with treatment and the feeding issues improved, allowing progressive discontinuation of nasogastric tube feeding. However, he remained corticosteroid-dependent (0.5–1 mg/kg/day), as attempts to wean him from prednisone resulted in recurrence of upper obstructive symptoms.

**Figure 4:**
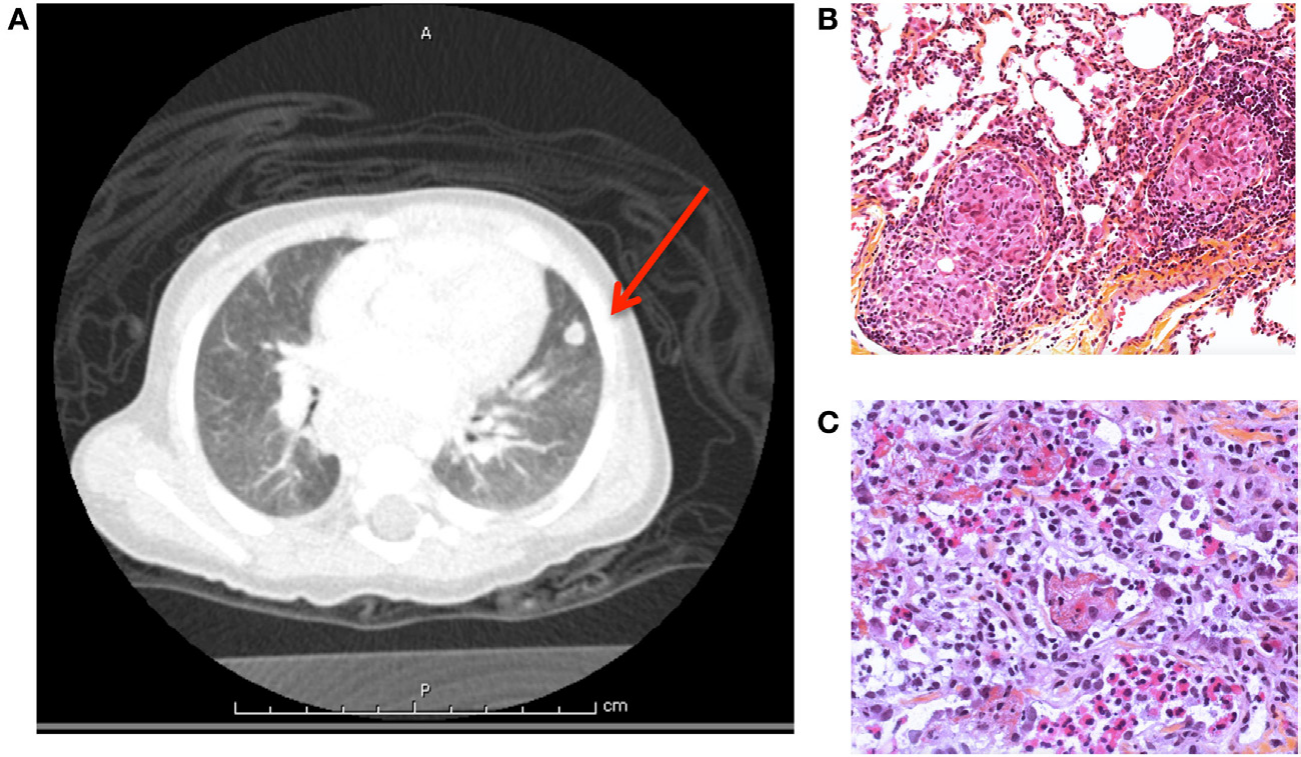
**(A)** Pulmonary computerized tomography scanner showed a nodule of the lingula, biopsy revealed *Mycobacterium avium* (case 1). Pulmonary biposy with presence of **(B)** granulomas and **(C)** eosinophilic infiltration.

During follow-up, he developed pulmonary nodules found on survey CT scan (Figure 4A). Culture from an open lung biopsy revealed the presence of *Mycobacterium avium* (Figures 4B,C). A treatment combining ethambutol, rifabutin, and azithromycin was initiated with favorable outcome.

The patient underwent bone marrow transplantation (BMT) with a matched unrelated donor (MUD) at 16 months of age, with a reduced intensity conditioning regimen consisting of busulfan, fludarabine, and alemtuzumab serotherapy. Mycophenolate mofetil and cyclosporine were used for graft-versus-host disease (GvHD) prophylaxis (1). He is currently 6 months post-BMT, and his evolution has been favorable with 90% donor chimerism and complete resolution of his symptoms.

### Case 2

The younger sibling of case 1, a male infant, was diagnosed with CGD at 48 h of life following a DHR flow cytometry assay that was performed given the positive family history (Figure 2). His older brother had been diagnosed 6 months earlier with CGD, and the mother who was already pregnant at the time of diagnosis, refused a prenatal diagnosis.

During the third trimester of pregnancy, antenatal US revealed polyhydramnios, bilateral hydronephrosis, a hyperechogenic gut, and ascites. He was born prematurely at 34 gestational weeks because of preterm labor, with a birth weight of 2,600 g (80th percentile). At birth, the child presented with a mild respiratory distress syndrome, necessitating less than 24 h of continuous positive airway pressure. Initial blood work showed major neutrophilia (37.18 × 10^9^/L) and eosinophilia (8.00 × 10^9^/L).

Post-natal renal US showed progressive resolution of the hydronephrosis during the first few weeks of life. He was however found to have unilateral grade V vesicourethral reflux, with evidence of a trabeculated bladder on a voiding cystourethrogram and bladder thickening was seen on US. There was no sign of obstruction on MAG-lasix evaluation. The patient remained asymptomatic with regard to these urological issues.

**Figure 5:**
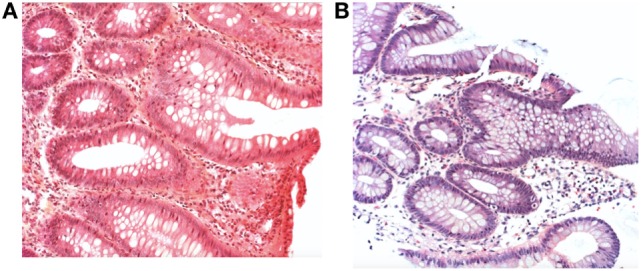
**(A)** Rectal and **(B)** colic biopsy revealing eosinophilic infiltration with H&E staining (case 2).

However, with initiation of feedings, he developed progressive abdominal distension with nausea and severe gastroesophageal reflux disease. He received an amino acid-based formula because of a suspicion of cow’s milk protein allergy, with only transient benefit. At 1 month of age, abdominal US revealed a pyloric thickening in the context of clinical deterioration with persistent vomiting and impaired growth parameters. Shortly after, he developed profuse hematochezia, and prednisone was initiated at a dose of 0.5 mg/kg. A rectal biopsy was performed and was compatible with an eosinophilic colitis (Figure 5).

At 6 weeks of life, while on treatment with prednisone, he had an acute deterioration with severe abdominal distension and bilious emesis, unresponsive to 1 mg/kg of IV methylprednisolone. An abdominal US showed diffuse thickening of the intestinal walls, predominantly at the antropyloric region, and a small bowel follow through showed parietal and mesenteric infiltration of the duodenum and jejunum (Figures 1E,F). Inflammatory markers were high with a CRP at 89.4 mg/L, absolute neutrophil count at 63.58 × 10^9^/L, and eosinophils at 15.74 × 10^9^/L (Table 1). Immunophenotyping revealed a hyperlymphocytosis of T, B, and NK cells, without evidence of excess activated HLA DR + T cells. The criteria for a secondary HLH were not met. After carefully excluding infectious causes, methylprednisolone was further increased at 2 mg/kg. Progressive improvement was noted over the following weeks, with upper and lower endoscopies performed 4 weeks later showing pyloric permeability and colonic mucosal improvement. The patient, however, presented with two further episodes of acute abdominal distension with intense discomfort and evidence of lower GI obstruction that were partially responsive to rectal enemas and Botox injection. The exact etiology of these subsequent episodes remains unclear. Despite his improvement with corticosteroids, the patient remained dependent on total parenteral nutrition (TPN) because of poor GI tolerance. Early impaired growth parameters improved after initiation of corticosteroids and resolved with transient TPN. He underwent BMT with a MUD at 5 months of age using the same conditioning regimen and GvHD prophylaxis as in case 1. TPN was weaned 2 months post-BMT, with a favorable clinical and laboratory evolution at 6 months post-BMT, and a donor chimerism of 86%. He does however remain on nasogastric feeds due to impaired acquisition of drinking skills, but he is improving rapidly and is now eating solid foods.

### Case 3

The third patient is a male infant who had an antenatal diagnosis of X-linked CGD, the mother being a known carrier. His *CYBB* mutation was identical to that of cases 1 and 2 (Figure 3), although his mother was from Polish origin. He was born at term with no complications. His DHR flow cytometry assay also showed a neutrophil stimulation index of 1 (Figure 2). Prophylaxis initially consisted of amoxicillin from birth followed by cotrimoxazole from 6 weeks, and itraconazole from 4 weeks. At 2 weeks of age, he presented with bloody diarrhea and abdominal discomfort. A cow’s milk protein allergy was then suspected, and because he refused hydrolyzed formula, breastfeeding was continued with exclusion of allergenic foods. Two weeks later, at 1 month of age, he was hospitalized for a recurrence of hematochezia and a cutaneous maculopapular rash. US and rectosigmoidoscopy findings were consistent with a recto-colitis, and biopsy revealed an inflammatory infiltrate of the recto-sigmoid with presence of many eosinophils. A mild hyperleukocytosis was noted with 19.5 × 10^9^/L leukocytes, and eosinophilia reaching 0.9 × 10^9^/L. Inflammatory markers were also elevated with a sedimentation rate of 50 mm/h and a CRP of 28.7 mg/L. Fecal calprotectin was 286 μg/g. Infectious causes for colitis were ruled out. Prednisolone 1 mg/kg was then started with rapid weaning over 5 weeks, with complete resolution of the colitis, leucocytosis, and inflammatory markers. His colitis has since then recurred episodically, although he did not require additional systemic corticosteroid treatments. This patient has not developed any growth failure.

At 5 months, multiple diffuse pulmonary nodules were found both on CT scan and PET scan during his pre-BMT workup. An open lung biopsy showed hyphae with granulomas suggestive of mold infection including aspergillosis, although a fungal PCR and culture were negative for fungal infection. Itraconazole was then switched for voriconazole, and while his imaging did not improve after 5 months of treatment, he remained clinically stable. He recently underwent BMT with a MUD following the same conditioning regimen and GvHD prophylaxis as cases 1 and 2.

## Background

Chronic granulomatous disease is a primary disease of phagocytic function caused by defects in subunits of the nicotinamide adenine dinucleotide phosphate (NADPH) oxidase, a membrane-bound enzyme that catalyzes the production of superoxide and promotes microbial killing (2, 3). Hemizygous mutations in the *CYBB* gene cause the X-linked form of CGD, which accounts for 2/3 of the cases of CGD, whereas biallelic pathogenic variants of either the *CYBA, NCF1, NCF2*, or *NCF4* genes lead to the autosomal recessive form of the disease (4).

Chronic granulomatous disease is characterized by variable degrees of immunodeficiency and dysregulation of inflammatory responses, leading to granulomas formation and inflammatory disease. Affected individuals present with recurrent and severe bacterial and fungal infections mostly involving the lungs, lymph nodes, liver, skin, and bones. The NADPH oxidase defect predisposes patients to infections with catalase positive organisms. The most frequently isolated pathogens include *S. aureus, Serratia marcescens, Burkholderia cepacia, Nocardia* spp., and fungi of the *Aspergillus* species (3, 5, 6). Inflammatory disease can present as a Crohn-like colitis (7), heterogeneous lung involvement (8), inflammation of the urinary tract (9), with granulomas predominantly affecting these organs and which can cause obstructive lesions (10). Inflammatory involvement of the GI tract is especially frequent in these patients, with up to 40% of patients developing GI symptoms throughout the illness (11, 12). When inflammation and granulomas occur, it is crucial to first rule out an infectious etiology. Corticosteroids are often used as first-line treatment for inflammatory and granulomatous complications. Prevention of infections with antibiotic and antifungal prophylaxis has significantly improved life expectancy (13). The only curative treatment to date is hematopoietic stem cell transplant, although gene therapy and gene editing are promising alternatives (14–17).

The X-linked form of CGD is considered to have a more severe clinical phenotype, which can be attributed to lower residual neutrophil-derived reactive oxygen intermediates production (18). In large series, the mean age at diagnosis was 3–5 years (5, 6). Although some very early-onset infectious manifestations have been described, inflammatory disease usually manifests later on. Here we report the unusual cases of severe gastric outlet obstruction (GOO) associated with eosinophilic colitis as a very early presentation of CGD in two male siblings, and a third unrelated patient with the same *CYBB* mutation presenting with eosinophilic colitis within the first weeks of life. All three patients had significant unexplained peripheral neutrophilia and eosinophilia, and very high inflammatory markers.

## Discussion

Chronic granulomatous disease is a particular immunodeficiency in which patients are commonly diagnosed early on, typically within their first decade of life (5, 6). Neonatal presentations have occasionally been described, but these usually occur in the context of infectious complications (19–21). These three patients presented with a very early-onset inflammatory bowel disease (in addition to a presumed eosinophilic cystitis for case 2), with evidence of systemic inflammation and marked hyperleukocytosis with peripheral eosinophilia. Moreover, the antenatal US findings for case 2 suggest that there might have been antenatal inflammatory obstructive lesions of the gut and bladder. To date, there has been only one report of CGD with antenatal manifestations, which presented as pericardial effusion with secondary hydrops fetalis (22). In a recent paper (23), Kawai et al. describe a cohort of patients with very early-onset CGD-associated colitis in which the youngest patient was 9 months old at diagnosis. Agarwal et al. have also reported a case of splenic and hepatic lesions in a febrile 40-day-old male infant with CGD suggestive of granulomas or abscesses (24). Urological disease is usually only apparent after 2 years of age in these patients (9, 25). It is also uncommon for CGD patients to present with multiple site inflammatory involvement, in particular so early as for case 2 (10). To our knowledge, this is the first case of neonatal CGD with such early-onset and severe symptoms of the gut and bladder in association with high inflammatory markers. GI tract involvement is more common among patients with the X-linked form, especially in the case of GOO (6, 12). Several reports have described GOO in CGD, but only Dickerman et al. and Varma et al. reported GOO as initial presentation in 2 infants at 17 and 20 months of age respectively (26, 27). To our knowledge, the patients we described are the youngest CGD patients reported who have had GOO occurring as the initial presentation. Altogether, the incidence of GOO as initial presentation remains unknown. In a limited cohort reported by Movahedi et al., 3.5% patients developed GOO during follow-up, but none presented it at diagnosis (11). This was similar to the results from the study by Cale et al. (28).

The peripheral neutrophilia and eosinophilia found in our patients is also atypical, especially in the absence of an infectious trigger. In Marciano et al.’s cohort, 17% of 140 patients with GI involvement presented with abnormal white blood cell counts. However, their range of 2.2–19.8 × 10^3^/μL white blood cells (WBC) is much lower than we observed in our cases (12). Eosinophilic inflammation has been reported in patients with CGD (25, 29, 30), mainly affecting the bladder and GI tract. In a series of 8 patients with CGD, colitis was characterized by eosinophilic crypt abscesses, pigmented macrophages, and a paucity of neutrophils (31). Recently, Nguyen et al. described the case of a 7-year-old boy with X-linked CGD that presented with a presumptive diagnosis of hypereosinophilic syndrome because of eosinophilic infiltration of the GI and genitourinary tracts with peripheral eosinophilia (32). Interestingly, similar to our patients, peripheral eosinophilia correlated with disease activity. The mechanism of inflammation and eosinophilia in CGD is poorly understood. Eosinophil major basic protein, the main constituent of the crystalloid core of the eosinophilic granule, has been shown to activate neutrophils by increasing NADPH oxidase activity (33). It has therefore been suggested that a deficient NADPH oxidase system could lead to an increased expression of the cytotoxic eosinophilic major basic protein *via* an unknown feedback mechanism that could lead to an eosinophilic inflammation seen in some CGD patients (29). However, this suggested mechanism does not account for the fact that only few CGD patients are described with eosinophilic inflammation or hypereosinophilia.

Lung infections are a common feature of CGD, arising in 80% of patients (34). However, atypical Mycobacteria have only been found in 2% of patients with pulmonary infections, based on published data from the USA (8). In a more recent extensive review of 71 CGD patients with mycobacterial disease, none had infections caused by environmental mycobacteria (35). The only pediatric case reported is that of a 10-month-old male with pulmonary infection to *M. avium* at the onset of CGD (36). The pulmonary infection to *M. avium* as in case 1 is thus another uncommon feature, especially at such a young age. Susceptibility to mycobacterial disease can be due to specific mutations in *CYBB* that affect the respiratory burst in macrophages (8). Finally, despite being usually of low pathogenicity in immunocompetent hosts, symptomatic infections to catalase-negative *Actinomyces* species have been reported in CGD patients (37), which is why we opted to treat our patient.

It is very remarkable that all three patients had the same *CYBB* mutation and it is tempting to speculate that these very unusual, early-onset and severe manifestations of CGD are the result of this particular genetic mutation. In the first family, because siblings (case 1 and 2) have a different father, a second genetic disease explaining this atypical presentation is unlikely. Moreover, case 3, which is unrelated to the first kindred and is from a different ethnical background, presented with a milder but similar phenotype, including a very early-onset colitis, with peripheral eosinophilia and high acute phase reactants, and a fungal lung infection within the first year. This mutation, which introduces a premature stop codon in exon 5, has already been described as pathogenic in the literature in correlation with a X91^0^ subtype, defined as an undetectable level of gp91-phox protein measured by immunoblot and/or spectral analysis. This subtype is predictive of a degree of severity (38, 39). Although no clear phenotype has been associated with this mutation, two other reported patients with this mutation were diagnosed by 5 months of age, thereby indicating early-onset manifestations of the disease (40). Nevertheless, many other nonsense mutations are associated with X91^0^ subtype and are not specifically described in very early-onset forms of the disease, nor with eosinophilia and severe inflammation. The link between this mutation and the phenotype we describe here has still to be confirmed.

## Concluding Remarks

We report the cases of three patients with X-linked CGD that presented with very early-onset inflammatory disease involving both the GI and urinary tracts, in addition to some evidence of antenatal involvement in one patient as well as systemic inflammation and extreme hyperleukocytosis with eosinophilia. To our knowledge, these exceptional cases are the first reported cases of CGD with such an early and severe presentation. This report also suggests that CGD diagnosis should be considered in patients with a persistent or chronic history of vomiting with features of GOO and/or evidence of eosinophilic inflammation.

## Ethics Statement

This study was carried out in accordance with the recommendations of “Good Clinical Practice” with written informed consent from all subjects. The protocol was approved by the “CHU Sainte-Justine ethic’s committee.”

## Author Contributions

RL and JA-D were responsible for data collection, data analyses, and for writing the manuscript. AB, GC, MLT, CD, MD, LF, PO, PT, FL, IF, FT, and HD were responsible for patient care and revision of the manuscript. UH and EH had primary responsibility of patient care and supervised the writing of the manuscript as senior authors.

## Conflict of Interest Statement

The authors declare that the research was conducted in the absence of any commercial or financial relationships that could be construed as a potential conflict of interest.

## References

[1] Gungor T Teira P Slatter M Stussi G Stepensky P Moshous D Reduced-intensity conditioning and HLA-matched haemopoietic stem-cell transplantation in patients with chronic granulomatous disease: a prospective multicentre study. Lancet (2014) 383(9915):436–48. 10.1016/S0140-6736(13)62069-3 24161820

[2] Segal AW. The function of the NADPH oxidase of phagocytes and its relationship to other NOXs in plants, invertebrates, and mammals. Int J Biochem Cell Biol (2008) 40(4):604–18. 10.1016/j.biocel.2007.10.003 18036868 PMC2636181

[3] Segal BH Leto TL Gallin JI Malech HL Holland SM. Genetic, biochemical, and clinical features of chronic granulomatous disease. Medicine (Baltimore) (2000) 79(3):170–200. 10.1097/00005792-200005000-00004 10844936

[4] Leiding JW Holland SM Chronic granulomatous disease. In: Pagon RA Adam MP Ardinger HH Wallace SE Amemiya A Bean LJH, editors. GeneReviews(R). Seattle, WA (1993). 22876374

[5] van den Berg JM van Koppen E Ahlin A Belohradsky BH Bernatowska E Corbeel L Chronic granulomatous disease: the European experience. PLoS One (2009) 4(4):e5234. 10.1371/journal.pone.0005234 19381301 PMC2668749

[6] Winkelstein JA Marino MC Johnston RB Jr Boyle J Curnutte J Gallin JI Chronic granulomatous disease. Report on a national registry of 368 patients. Medicine (Baltimore) (2000) 79(3):155–69. 10.1097/00005792-200005000-00003 10844935

[7] Marks DJ Miyagi K Rahman FZ Novelli M Bloom SL Segal AW. Inflammatory bowel disease in CGD reproduces the clinicopathological features of Crohn’s disease. Am J Gastroenterol (2009) 104(1):117–24. 10.1038/ajg.2008.72 19098859

[8] Mahdaviani SA Mohajerani SA Rezaei N Casanova JL Mansouri SD Velayati AA. Pulmonary manifestations of chronic granulomatous disease. Expert Rev Clin Immunol (2013) 9(2):153–60. 10.1586/eci.12.98 23390946

[9] Walther MM Malech H Berman A Choyke P Venzon DJ Linehan WM The urological manifestations of chronic granulomatous disease. J Urol (1992) 147(5):1314–8. 10.1016/S0022-5347(17)37552-3 1569675

[10] Magnani A Brosselin P Beaute J de Vergnes N Mouy R Debre M Inflammatory manifestations in a single-center cohort of patients with chronic granulomatous disease. J Allergy Clin Immunol (2014) 134(3):655–62.e8. 10.1016/j.jaci.2014.04.014 24985400

[11] Movahedi M Aghamohammadi A Rezaei N Farhoudi A Pourpak Z Moin M Gastrointestinal manifestations of patients with chronic granulomatous disease. Iran J Allergy Asthma Immunol (2004) 3(2):83–7.03.02/ijaai.8387 17301397

[12] Marciano BE Rosenzweig SD Kleiner DE Anderson VL Darnell DN Anaya-O’Brien S Gastrointestinal involvement in chronic granulomatous disease. Pediatrics (2004) 114(2):462–8. 10.1542/peds.114.2.462 15286231

[13] Gallin JI Alling DW Malech HL Wesley R Koziol D Marciano B Itraconazole to prevent fungal infections in chronic granulomatous disease. N Engl J Med (2003) 348(24):2416–22. 10.1056/NEJMoa021931 12802027

[14] Seger RA. Chronic granulomatous disease: recent advances in pathophysiology and treatment. Neth J Med (2010) 68(11):334–40. 21116026

[15] De Ravin SS Li L Wu X Choi U Allen C Koontz S CRISPR-Cas9 gene repair of hematopoietic stem cells from patients with X-linked chronic granulomatous disease. Sci Transl Med (2017) 9(372):eaah3480. 10.1126/scitranslmed.aah3480 28077679

[16] Kohn DB Kuo CY. New frontiers in the therapy of primary immunodeficiency: from gene addition to gene editing. J Allergy Clin Immunol (2017) 139(3):726–32. 10.1016/j.jaci.2017.01.007 28270364 PMC5911283

[17] Kaufmann KB Chiriaco M Siler U Finocchi A Reichenbach J Stein S Gene therapy for chronic granulomatous disease: current status and future perspectives. Curr Gene Ther (2014) 14(6):447–60. 10.2174/1566523214666140918113201 25245086

[18] Kuhns DB Alvord WG Heller T Feld JJ Pike KM Marciano BE Residual NADPH oxidase and survival in chronic granulomatous disease. N Engl J Med (2010) 363(27):2600–10. 10.1056/NEJMoa1007097 21190454 PMC3069846

[19] Mouy R Ropert JC Donadieu J Hubert P de Blic J Revillon Y [Chronic septic granulomatosis revealed by neonatal pulmonary aspergillosis]. Arch Pediatr (1995) 2(9):861–4. 10.1016/0929-693X(96)81264-4 7581783

[20] Theobald I Fischbach R Hulskamp G Franzius C Frosch M Roth J [Pulmonary aspergillosis as initial manifestation of septic granulomatosis (chronic granulomatous disease, CGD) in a premature monozygotic female twin and FDG-PET diagnosis of spread of the disease]. Radiologe (2002) 42(1):42–5. 10.1007/s117-002-8116-0 11930540

[21] Saito S Oda A Kasai M Minami K Nagumo H Shiohara M A neonatal case of chronic granulomatous disease, initially presented with invasive pulmonary aspergillosis. J Infect Chemother (2014) 20(3):220–3. 10.1016/j.jiac.2013.10.008 24674387

[22] Michailidis GD Hourihane JO Sievers R O’Donnell AI Howe DT In-utero pericardiocentesis to treat fetal hydrops caused by X-linked chronic granulomatous disease. Ultrasound Obstet Gynecol (2006) 28(1):117–9. 10.1002/uog.2831 16795136

[23] Kawai T Arai K Harayama S Nakazawa Y Goto F Maekawa T Severe and rapid progression in very early-onset chronic granulomatous disease-associated colitis. J Clin Immunol (2015) 35(6):583–8. 10.1007/s10875-015-0180-2 26233238

[24] Agarwal S. Chronic granulomatous disease. J Clin Diagn Res (2015) 9(5):SD01–2. 10.7860/JCDR/2015/12139.5945 26155526 PMC4484118

[25] Claps A Della Corte M Gerocarni Nappo S Francalanci P Palma P Finocchi A How should eosinophilic cystitis be treated in patients with chronic granulomatous disease? Pediatr Nephrol (2014) 29(11):2229–33. 10.1007/s00467-014-2883-7 25037864

[26] Dickerman JD Colletti RB Tampas JP. Gastric outlet obstruction in chronic granulomatous disease of childhood. Am J Dis Child (1986) 140(6):567–70. 3706237 10.1001/archpedi.1986.02140200077032

[27] Varma VA Sessions JT Kahn LB Lipper S. Chronic granulomatous disease of childhood presenting as gastric outlet obstruction. Am J Surg Pathol (1982) 6(7):673–6. 10.1097/00000478-198210000-00009 7180966

[28] Cale CM Jones AM Goldblatt D. Follow up of patients with chronic granulomatous disease diagnosed since 1990. Clin Exp Immunol (2000) 120(2):351–5. 10.1046/j.1365-2249.2000.01234.x 10792387 PMC1905649

[29] Jaggi P Freeman AF Katz BZ. Chronic granulomatous disease presenting with eosinophilic inflammation. Pediatr Infect Dis J (2005) 24(11):1020–1. 10.1097/01.inf.0000183775.69035.33 16282946

[30] Barese CN Podesta M Litvak E Villa M Rivas EM Recurrent eosinophilic cystitis in a child with chronic granulomatous disease. J Pediatr Hematol Oncol (2004) 26(3):209–12. 10.1097/00043426-200403000-00014 15125617

[31] Schappi MG Klein NJ Lindley KJ Rampling D Smith VV Goldblatt D The nature of colitis in chronic granulomatous disease. J Pediatr Gastroenterol Nutr (2003) 36(5):623–31. 10.1097/00005176-200305000-00006 12717086

[32] Nguyen A Patel K Puck J Dorsey M Longstanding eosinophilia in a case of late diagnosis chronic granulomatous disease. J Clin Immunol (2017) 37(2):101–3. 10.1007/s10875-016-0361-7 27966181

[33] Moy JN Gleich GJ Thomas LL. Noncytotoxic activation of neutrophils by eosinophil granule major basic protein. Effect on superoxide anion generation and lysosomal enzyme release. J Immunol (1990) 145(8):2626–32; quiz 792–3. 2170521

[34] Towbin AJ Chaves I. Chronic granulomatous disease. Pediatr Radiol (2010) 40(5):657–68. 10.1007/s00247-009-1503-3 20135113

[35] Conti F Lugo-Reyes SO Blancas Galicia L He J Aksu G Borges de Oliveira E Jr Mycobacterial disease in patients with chronic granulomatous disease: a retrospective analysis of 71 cases. J Allergy Clin Immunol (2016) 138(1):241–8.e3. 10.1016/j.jaci.2015.11.041 26936803

[36] Ohga S Ikeuchi K Kadoya R Okada K Miyazaki C Suita S Intrapulmonary *Mycobacterium avium* infection as the first manifestation of chronic granulomatous disease. J Infect (1997) 34(2):147–50. 10.1016/S0163-4453(97)92509-3 9138139

[37] Reichenbach J Lopatin U Mahlaoui N Beovic B Siler U Zbinden R Actinomyces in chronic granulomatous disease: an emerging and unanticipated pathogen. Clin Infect Dis (2009) 49(11):1703–10. 10.1086/647945 19874205 PMC4100544

[38] Roos D Kuhns DB Maddalena A Roesler J Lopez JA Ariga T Hematologically important mutations: X-linked chronic granulomatous disease (third update). Blood Cells Mol Dis (2010) 45(3):246–65. 10.1016/j.bcmd.2010.07.012 20729109 PMC4360070

[39] Stasia MJ Bordigoni P Floret D Brion JP Bost-Bru C Michel G Characterization of six novel mutations in the CYBB gene leading to different sub-types of X-linked chronic granulomatous disease. Hum Genet (2005) 116(1–2):72–82. 10.1007/s00439-004-1208-5 15538631

[40] Kannengiesser C Gerard B El Benna J Henri D Kroviarski Y Chollet-Martin S Molecular epidemiology of chronic granulomatous disease in a series of 80 kindreds: identification of 31 novel mutations. Hum Mutat (2008) 29(9):E132–49. 10.1002/humu.20820 18546332

